# Evolutionary History of a Desert Shrub *Ephedra przewalskii* (Ephedraceae): Allopatric Divergence and Range Shifts in Northwestern China

**DOI:** 10.1371/journal.pone.0158284

**Published:** 2016-06-28

**Authors:** Zhi-Hao Su, Ming-Li Zhang

**Affiliations:** 1 Key Laboratory of Biogeography and Bioresource in Arid Land, Xinjiang Institute of Ecology and Geography, Chinese Academy of Sciences, Urumqi, 830011, China; 2 Institute of Botany, Chinese Academy of Sciences, Beijing, 100093, China; Technical University in Zvolen, SLOVAKIA

## Abstract

Based on two chloroplast DNA sequences, *psb*A*-trn*H and *trn*T*-trn*F, phylogeographical patterns of a desert shrub, *Ephedra przewalskii*, were examined across most of its geographic range in northwestern China. A total of sixteen haplotypes were detected. There was a common haplotype in each basin, that was haplotype A in Tarim Basin, haplotype G in Junggar Basin, and haplotype M in Qaidam Basin. Genetic variance mainly occurred among populations, geographic regions, and eleven geographic groups subdivided by SAMOVA analysis. *E*. *przewalskii* likely had a smaller and more fragmented geographic range during the Last Glacial Maximum, which was determined based on ecological niche modelling. Three groups of *E*. *przewalskii* populations were detected to have experience range expansion, and this was based on significant values of Fu’s F_S_, Tajima’s D, and unimodel mismatch distributions. The cold and dry climate during the glacial period of the Quaternary is postulated to have been a driver for significant genetic isolation and divergence among populations or groups in *E*. *przewalskii*, whereas the warmer and wetter climate during the interglacial period is speculated to have provided favourable conditions for range expansion of the species.

## Introduction

The vast arid northwestern China comprises the entire Xinjiang Province, Hexi Corridor, Qaidam Basin, and western parts of Helan Mountains in Inner Mongolia (Alxa Dersert) [1]. In this region, Tarim Basin is the biggest endorheic basin, covering approximately 530,000 km^2^, and it is surrounded by Tianshan Mountains on the north, Kunlun and Altun Mountains on the south, Pamir Plateau on the west, and the east rim opens to Hexi Corridor. Junggar Basin, which is the second biggest interior basin and covers approximately 380,000 km^2^, is surrounded by Tianshan Mountains on the south, Altai Mountains on the north, and Alatau and Tarbagatai Mountains on the west. Junggar Basin is separated from Tarim Basin by Tianshan Mountains. Hami Basin locates in the east of Tianshan Mountains, and connects with Tarim Basin, Junggar Basin, and Hexi Corridor. Qaidam Basin, the third biggest interior basin in northwestern China, covers ca. 250,000 km^2^ and is relatively closed and surrounded by Kunlun Mountains in the south, Altun Mountains in the northwest, and Qilian Mountains in the north.

In arid northwestern China, most plants groups are relicts, ancient, single-species or few-species, and endemic to the gobi (stony deserts) and deserts. Desert species such as *Ammopiptanthus mongolicus* (Masxim. ex Kom.) S.H. Cheng, *Tetraena mongolica* Maxim., and *Ephedra przewalskii* Stapf, are typical elements of the flora. In the Quaternary, pollen records provide evidence that glaciation was not present in deserts of the region, except in the intervening mountain ranges [2,3]. However, climate oscillations have had profound effects on the evolutionary processes of native desert species [4,5]. This has resulted in allopatric divergences [6,7], regional range expansion [8,9], and contraction or fragmentation of population distribution [5].

*Ephedra przewalskii* is a drought-tolerant shrub inhabiting extremely dry semi-deserts, piedmont, and gravelly saline soil, usually forming a large area within the plant community. It is usually an indicator species in plant communities of the deserts in northwestern China, and due to its powerful resistance to drought and sand burial, it is commonly used as a sand binder [10]. It is primarily restricted to the western Inner Mongolia (Alxa Desert), Hexi Corridor, and the three major endorheic basins of China. It also extends west to Kazakhstan, Kyrgyzstan, Pakistan, Tajikistan, Uzbekistan, and extends north to Mongolia [10].

The phylogeographical history of *Ephedra* species distributed in Qinghai-Tibet-Plateau (QTP) and adjacent arid regions has been studied, and three main lineages were found and systematic positions of some species were confirmed among members of the genus [11]. However, for *E*. *przewalskii*, an ancient species with an extensive geographic range, the information concerning genetic structure and phylogeography patterns across the whole distribution range in China was still unclear, and the following questions concerning this species have not been investigated: 1) genetic differences among the populations, 2) allopatric divergence among the basins, 3) and detection of range expansion throughout the species. These questions will provide a greater better knowledge to out understanding of the evolutionary history of plants in arid northwestern China.

Chloroplast DNA (cpDNA) in plants is inherited maternally, and evolves slowly with low recombination and mutation rates [12,13], so it can be treated as a single haplotype in phylogeographical analyses [14,15,16]. Moreover, variation in cpDNA loci is often more geographically structured than that of nuclear loci [17]. In addition, population historical dynamic of species can be predicted by ecological niche models (ENMs) [18,19,20]. Thus, we attempted to combine chloroplast phylogeography with ENMs, to address the following questions: 1) quantify levels of genetic variation within and among populations, 2) examine if geographical distribution pattern of genetic variation was influenced by climate fluctuations in the Pleistocene, and, if so, how?

## Materials and Methods

### Sampling

*Ephedra przewalskii* has woody stems, and is usually 0.5–2.4 meters high. The cone is usually sessile, and crowded into a group, opposite or whorled on the knot. The male cone is hazel, nearly spherical, with 7–8 stamens. The unique morphological feature of *E*. *przewalskii*, by which it differs from the other species of *Ephedra* in China, is that, when female cone mature, the bracts are increscent but not fleshy, dry, colourless, semi-lucent, and membranous. We sampled a total of 469 individuals from 45 populations, covering almost the entire geographic range of the species in China. Of the sampled populations, one (population 1) was from Urumqi, one (population 2) was from Turpan Basin, nineteen (populations 3–21) were from Tarim Basin, eight (populations 22–29) were from Junggar Basin, one (population 30) was from Hami Basin, seven (populations 31–33, 36–38, and 45) were from Hexi Corridor, two (populations 34 and 35) were from Alxa Desert of Inner Mongolia, and six (populations 39–44) were from Qaidam Basin (S1 Table). In each population, 10 to 12 individuals were collected and, from each, fresh stems were gathered and dried in silica gel.

Our study did not concern Human Subject Research or Animal Research. We can confirm that all sample locations of field collection are public, whether national park nor other protected area of land, thus the collections are permissible, and the stem materials collected are not the endangered or protected species.

### DNA extraction, amplification, and sequencing

Total genomic DNA was extracted from dried leaf tissue using a modified 2 × CTAB method [21,22]. After screening several gene segments, we chose two cpDNA non-coding spacers, *trn*H–*psb*A and *trn*T–*trn*F, to conduct our study. The two spacers were amplified and sequenced using the primers and protocols of Sang et al. (1997) and Taberlet et al. (1991) respectively [23,24]. Polymerase chain reaction (PCR) amplification for the two markers was performed using the reaction mix as follows: 2 mM MgCl_2_, 200 mM dNTP, 1 pmol primer, and 0.025 U mL^-1^ Taq polymerase (Takara), and the temperature profile of the amplification process is: 95°C for 1 min; 30 cycles of 94°C for 30 s; 52°C for 30 s; and 72°C for 1 min 30 s linked to 72°C for 10 min. Forward and reverse primers of the PCR amplification were also used in sequencing, conducted by the DYEnamic ET Terminator Kit (Amersham Pharmacia Biotech) at the Shanghai Sangon Biological Engineering Technology & Services Co., Ltd (Shanghai, China) with the use of an ABI-PRISM 3730 automatic DNA analyzer. Electropherograms were edited and assembled using SEQUENCHER, version 4.8. Sequences were aligned with CLUSTAL W [25] and refined by visual inspection.

### Phylogeographical analysis and divergence time estimate

Phylogenetic analysis of the resulting alignments was carried out by maximum-likelihood (ML) in PAUP* version 4.0b10 [26], to investigate the phylogenetic relationships of the cpDNA haplotypes. Characters were weighted equally and their state changes were treated as unordered. We used Modeltest 3.7 [27] to search for the best nucleotide substitution model. The reliability of the ML trees was tested using 1000 bootstrapping replicates. We also used BEAST [28,29] to conduct Bayesian analyses to search for tree topologies and estimate divergence times between the lineages. In BEAST, we used a constant-size coalescent tree prior and the HKY substitution model in the analysis. Each indel was treated as a single mutation event and coded as a substitution in the Bayesian analysis [30]. The cpDNA substitution rates of most angiosperm species were estimated to vary between 1 and 3×10^−9^ substitutions per site per year (s/s/y) [31]. Because of the uncertainty of the rates, we used normal distribution priors with a mean of 2×10^−9^ and a SD of 6.080×10^−10^ within the 95% range of the distribution to estimate the divergence times [32]. All parameters were sampled once every 1000 steps from 10 000 000 Markov chain Monte Carlo steps, after a burn-in of 1 000 000 steps. By visual inspection of plotted posterior estimates, convergence of the stationary distribution was examined using TRACER [29], and the effective sample size for each parameter sampled was found to be over 200. A network of all haplotypes was constructed using statistical parsimony [33] implemented in the program TCS v. 1.21, with a maximum connection limit equal to 14 steps [34]. For the phylogenetic analysis, we chose *Ephedra monosperma* and *E*. *major* as the outgroups of haplotypes of *E*. *przewalskii*, and for the network, we used *E*. *monosperma* as the outgroup.

### Molecular variability and demographic analysis

Within-population diversity (*H*_S_), total gene diversity (*H*_T_), genetic differentiation (*G*_ST_), and population subdivision of phylogenetically ordered alleles (*N*_ST_) were estimated using HAPLONST. Spatial analysis of molecular variance was performed using SAMOVA, version 1.0, to test the spatial genetic structure, defining groups that are geographically homogeneous and genetically differentiated [35]. The analysis was run from *K* = 2 to 44, with 100 random initial conditions for each run. Analyses of molecular variance (AMOVA) at different levels were performed to study the genetic structure of the populations [36].

Tajima’s *D*, Fu’s *F*_*S*_, and the mismatch distribution were calculated to test for range expansions [37,38,39,40]. A significant value for *D* or a significant, large negative value for *F*_*S*_, or an unimodal shape of the mismatch distribution may be the result of population expansion [38,41,42,43,44]. All expansion tests were performed in ARLEQUIN, version 3.01 [44], with 10 000 permutations to test for significance. If the sudden expansion model was detected, we then used the relationship *τ* = 2*ut* to calculate the expansion time (*t*) [44], where *τ* is the total number of mutations, and *u* is the mutation rate per generation for the whole analyzed sequence. The value of *u* was calculated as *u* = 2 *μ*·*k*·*g*, where *μ* is the substitution rate per nucleotide site per year (s /s/y), *k* is the mean sequence length of the analyzed DNA region, and *g* is the generation time in years. We again used a mean substitution rate of 2.0 × 10^−9^ to estimate the expansion times of SAMOVA groups. According to our initial observations, we assumed the generation time of this desert species to be 4 years.

### Present and past distribution modeling

**Fig 1:**
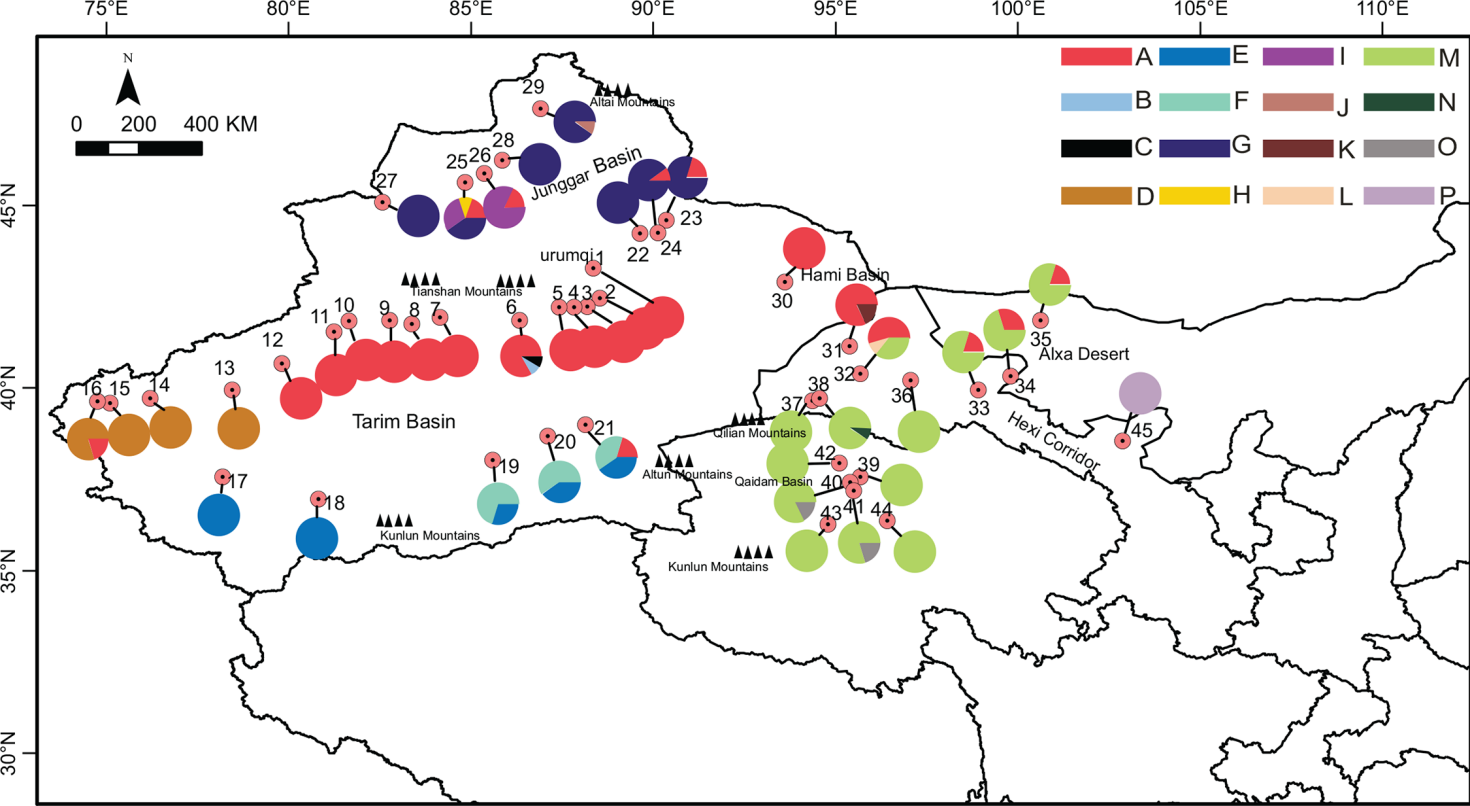
Geographical distribution of *Ephedra przewalskii* in China, population numbers and haplotypes of *E*. *przewalskii* correspond to those in S1 Table and S2 Table respectively, pie-charts represent haplotype frequency. Xinjiang Province includes Urumqi, Tarim basin, Junggar basin, and Hami basin (i.e. populations 1–30).

Based on the geographical locations of the *Ephedra przewalskii* populations included in this study (Fig 1, S1 Table), ENMs were carried out in MAXENT, version 3.2.1 [18] to predict potentially suitable past (Last Glacial Maximum; LGM) and present climate envelopes for the species. Geographical distributions was modeled for the present using the WorldClim dataset [45], and for the past, (LGM, approximately 21 kya before present), using the Community Climate System Model [46]. The climatic niche of the species was modelled using eight (out of 19) BIOCLIM variables screened by principal component analysis. These variables were the mean annual temperature, mean diurnal range, temperature seasonality, mean temperature of driest quarter, mean temperature of coldest quarter, annual precipitation, precipitation of the driest month and precipitation seasonality. This restricted dataset was used to avoid including highly correlated variables and prevent potential overfitting [47]. We performed ENMs using the default parameters of MAXENT and the user-selected features: regularization multiplier of 3.0, application of a random seed, duplicate presence records removal, and logistic probabilities used for output [48]. The area under the receiver operating characteristic curve (AUC) was calculated to evaluate model performance. We used 75% of localities to train the model and 25% to test the model. Values between 0.7 and 0.9 indicate good discrimination [49].

## Results

### Sequence analysis

For the aligned *trn*H–*psb*A spacer, the length was 473 base pairs (bp), and for the *trn*T–*trn*F spacer, the length was 656 bp. A total of 12 informative characters were detected in the aligned sequence data: 7 nucleotide substitutions (positions 443, 467, 493, 596, 730, 996, and 1032) and 5 indels (positions 424, 470, 1093, 1094, and 1117). From the 469 sampled individuals of 45 populations, a total of 16 haplotypes (A–P) were identified (S1 Table; S2 Table). GenBank accession numbers of the cpDNA sequences are KR733301—KR733316.

### Haplotype geographical distribution and relationships

The geographic coordinates and the frequency of cpDNA haplotypes in each population, were presented in Fig 1 and S1 Table. Haplotype A was widespread in the north of Tarim Basin, Urumqi, and Turpan Basin, and beyond Hami Basin, it extended north to Junggar Basin and south to Hexi Corridor. Haplotype D was only found in the west of Tarim Basin. Haplotype E and F were found in the south of the basin, and merged with haplotype A in the east of the basin. Haplotype G was widespread in Junggar Basin, and some other rare haplotypes, such as H, I and J, were also found in the basin. Haplotype M was widespread in Qaidam Basin and its adjacent areas, and haplotype O was distributed in the center of the basin. Haplotypes A and M merged in the north of Hexi corridor and extended to Alxa Desert, whereas haplotype P was found 400 kms away in the south of Hexi corridor (population 45).

**Fig 2:**
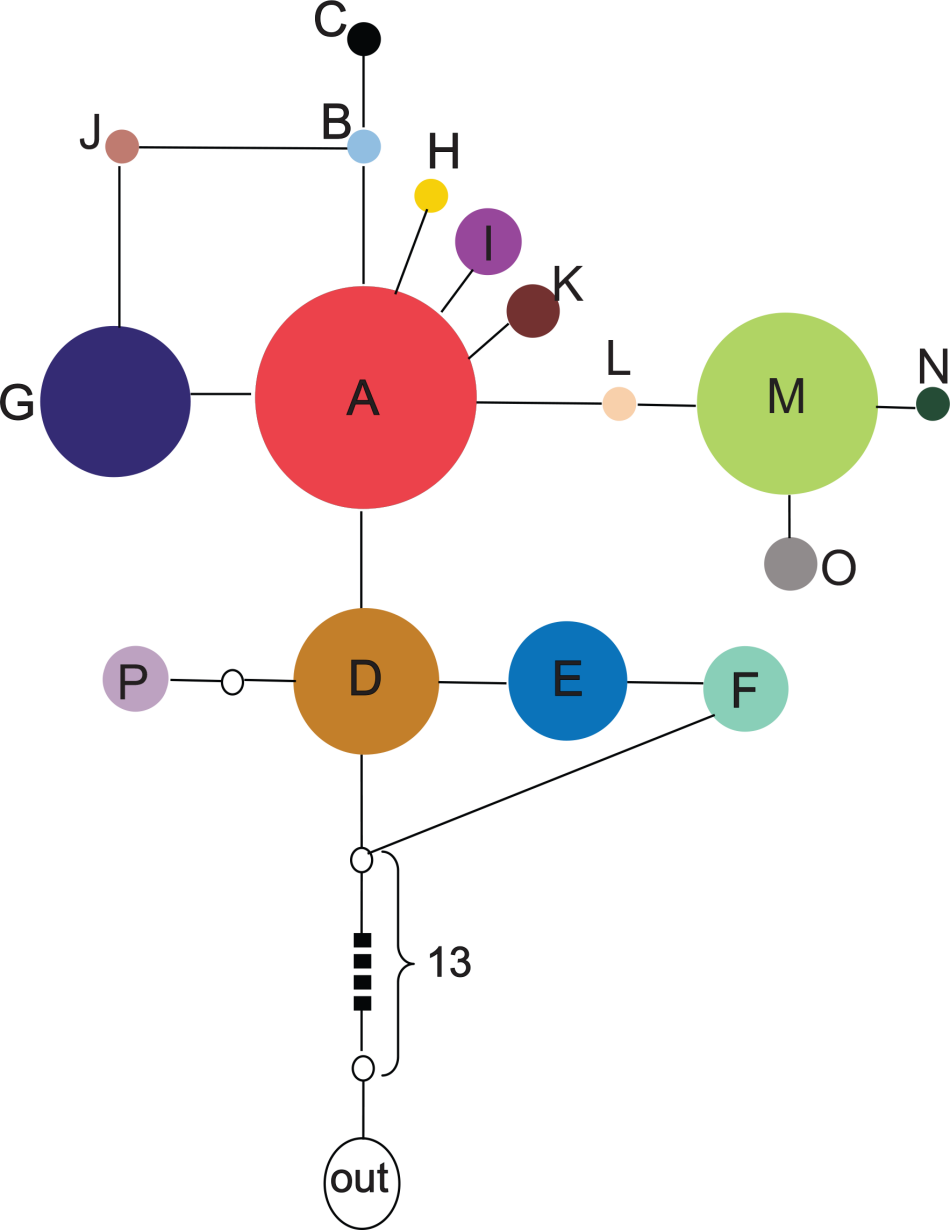
Statistical parsimony network of *Ephedra przewalskii* cpDNA haplotypes. The unfilled circles indicate the missing or inferred haplotypes; the circle size is proportional to haplotype frequencies; haplotypes in the network showed in the same colours correspond to those in the geographical distribution, Fig 1.

In the network (Fig 2), haplotypes D, E and F were in a centra position and therefore presumably ancestral. Haplotypes A was connected to D by one substitution, and haplotype G was connected to A by one substitution. Haplotype L was connected to A by one substitution, which in turn was connected to M by another substitution. Most haplotypes were arranged in the network as would be expected from their geographic arrangement. The exception was haplotype P located further in the south of Hexi corridor. Haplotype P was closely associated with haplotype D, located in the west of Tarim Basin.

### Genetic diversity and genetic structure

In the spatial analysis of molecular variance, *F*_*CT*_ values increased to a maximum of 0.8799 when the number of groups (*K*) was raised from 2 to 11. The subdivision pattern of populations corresponding to *K* = 11 was represented in Table 1.

**Table 1:** Results of spatial analysis of molecular variance corresponding to *K* = 11.

Groups	Populations and regions
1	1 (Urumqi), 2 (Turpan Basin), 3–12 (Tarim Basin), 25 (Junggar Basin), 30 (Hami Basin), 31 (Hexi Corridor)
2	13–16 (Tarim Basin)
3	17–18 (Tarim Basin)
4	19–20 (Tarim Basin)
5	21 (Tarim Basin)
6	22–24, 27–29 (Junggar Basin)
7	26 (Junggar Basin)
8	32 (Hexi Corridor)
9	33 (Hexi Corridor), 34-35(Alxa Desert)
10	36–38 (Hexi Corridor), 39–44 (Qaidam Basin)
11	45 (Hexi Corridor)

Within-population gene diversity (*H*_S_) was 0.161 (SE 0.0335), and total gene diversity (*H*_*T*_) was 0.800 (*SE* 0.0342). Differentiation among populations was high (*G*_*ST*_ = 0.799, *SE* 0.0408), indicating populations were highly structured. *N*_*ST*_ (0.857, *SE* 0.0318) was significantly higher than *G*_*ST*_ as shown by the U-test (*U* = 3.60, *P* < 0.01), demonstrating a significant phylogeographic structure. AMOVA analysis showed that 86.21% (*P*<0.001) of the total variation occurred among populations, 46.05% (*P*<0.001) of that occurred among geographical regions, and 87.99% (*P*<0.001) of that occurred among SAMOVA groups (Table 2).

**Table 2:** Results of analysis of molecular variance for *Ephedra przewalskii* based on chloroplast DNA sequence data.

Source of variation	d.f.	Sum of squares	Variance components	Percentage of variation (%)
Among populations	44	318.227	0.6835	86.21*
Within populations	424	46.374	0.1094	13.79
(1–12, 25, 30–31) vs. (13–16) vs. (17–18) vs. (19–20) vs. (21) vs. (22–24, 27–29) vs. (26) vs. (32) vs. (33–35) vs. (36–44) vs. (45)				
Among groups	10	313.467	0.8228	87.99*
Among populations within groups	34	4.760	0.0029	0.31*
Within populations	424	46.374	0.1094	11.70*
Geographic regions				
Among geographic regions	7	170.552	0.4103	46.05*
Among populations within regions	37	147.675	0.3713	41.68*
Within populations	424	46.374	0.1094	12.17*

* *P* < 0.001

### Demography of the groups in *Ephedra przewalskii*

Statistics of Fu’s *F*_*S*_, Tajiam’s *D*, and the mismatch distribution were analysed for every subdivided group, and the results showed that significant values of Fu’s *F*_*S*_, Tajiam’s *D*, and unimodal distributions of the mismatch distribution were detected in groups 1, 6, and 10, and the total samples (S1 Fig; Table 3); thus, these groups and the total samples were speculated to have experienced recent range expansion in the past. The range expansion time of the groups was estimated to have occurred at approximately 83 Kya, and that of the total samples was approximately 53 Kya, which was consistent with the deglaciation period in the Last Interglaciation and the Interstadial of The Last Glaciation respectively [50].

**Table 3:** Results of neutrality tests and mismatch distribution analysis for *Ephedra przewalskii*.

Group	*τ*	SSD (*P* value)	Hrag (*P* value)	Tajiam’s *D* (*P* value)	Fu ‘s *Fs* (*P* value)
Group (1)	3.00	0.0000 (0.39)	0.5406 (0.68)	-1.3498 (0.043)	-8.5975 (0.000)
Group (6)	3.00	0.0002 (0.39)	0.5817(0.65)	-0.7136 (0.235)	-2.1094 (0.011)
Group (10)	3.00	0.0001 (0.33)	0.6491 (0.68)	-1.0324 (0.11)	-2.3196 (0.012)
Total	1.914	0.0105 (0.13)	0.0485 (0.38)	0.5988 (0.76)	-3.9892 (0.118)

*τ*: time in number of generations elapsed since the sudden expansion episode; Hrag: the Harpending’s Raggedness index; SSD: sum of squared deviations.

### Phylogenetic analysis and divergence time

**Fig 3:**
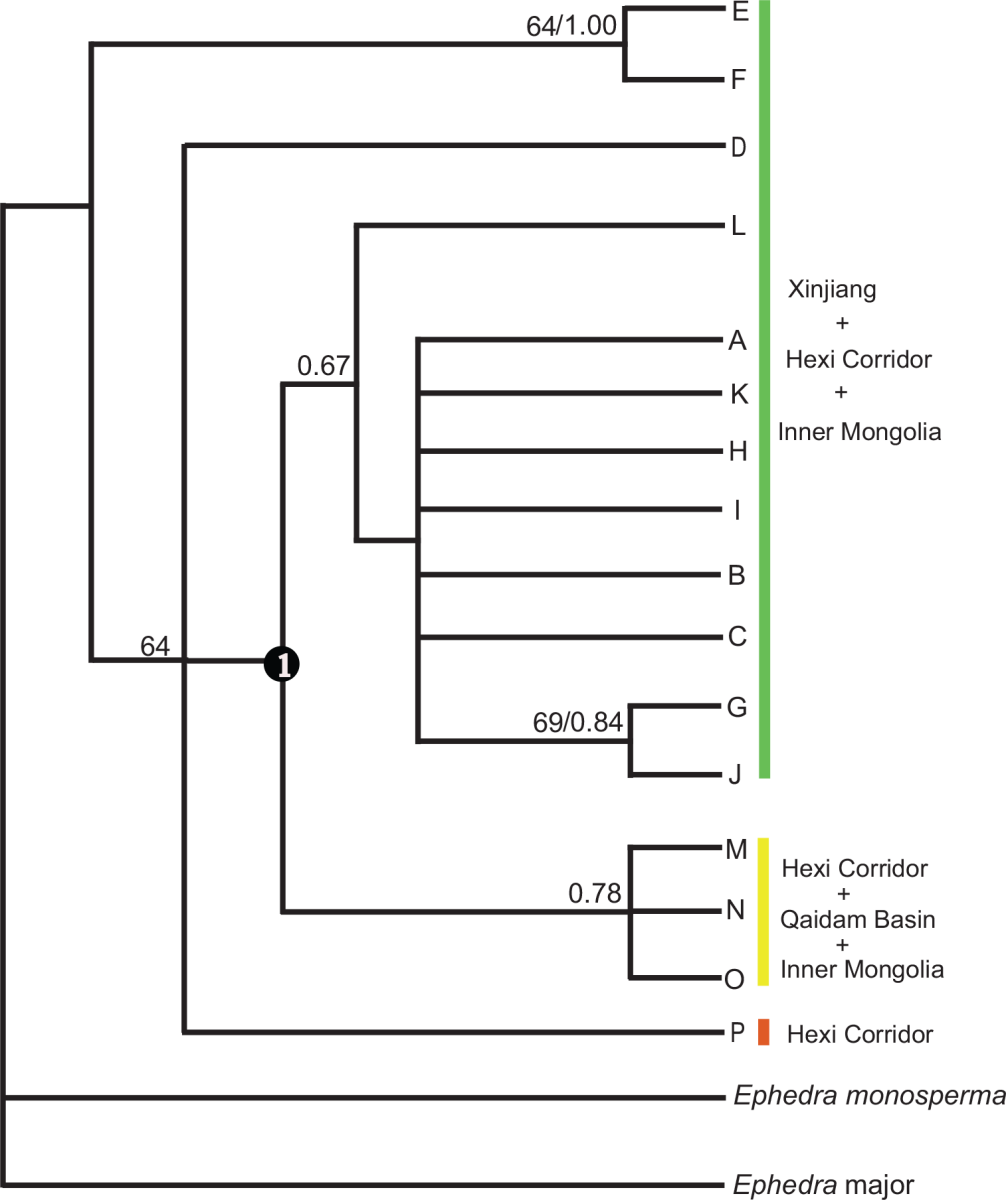
Phylogenetic relationships of 16 haplotypes of *Ephedra przewalskii* and related species. Numbers above branches are support values (support values > 0.50).

Trees produced by ML and Bayesian analyses had the same topology, and thus only the ML tree was presented (Fig 3). In this tree, *Ephedra przewalskii* was resolved as monophyletic and included two main clades. The first clade consisted of haplotypes E and F with high support values (64%; 1.00), that was sister to the second clade including all other haplotypes except E and F, with a moderate support value (64%). The second clade consisted of an inner monophyletic clade (node 1, 51%) and two paraphyletic clades, haplotype D and P. In the inner monophyletic clade, haplotypes A, B, C, G, H, I, J, K, and L clustered into a clade with a moderate support value 0.67, and haplotypes M, N, and O clustered into a clade with a moderate support value 0.78. Divergence times at node 1 was estimated at 0.73 Mya (early-middle Pleistocene).

### Past and present distribution of *Ephedra przewalskii*

**Fig 4:**
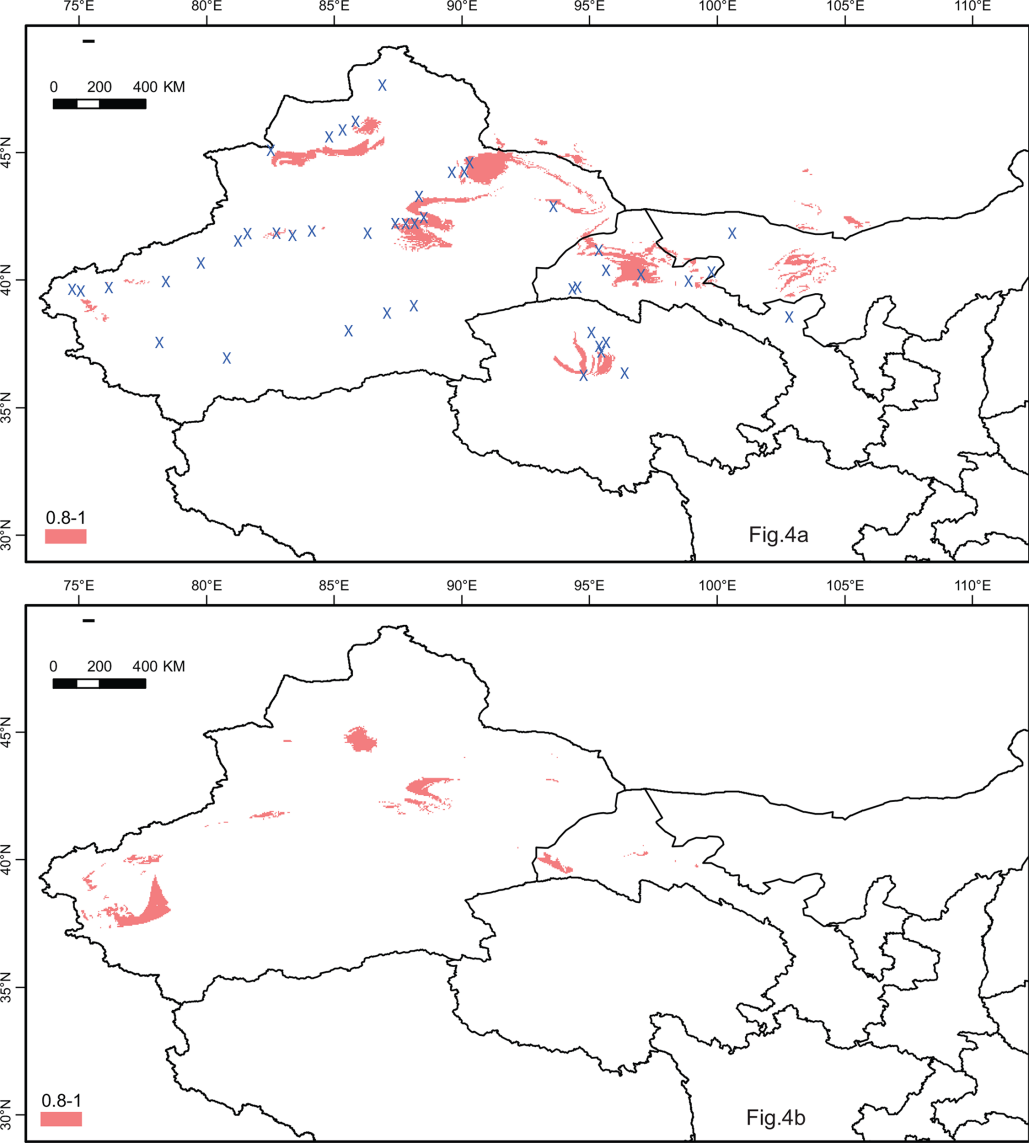
Maps depicting potential distribution as occurrence probability of *Ephedra przewalskii* in the northwestern China during (a) present-day and (b) LGM based on the CCSM models as derived from the ecological niche models (ENMs) in MAXENT.

The test AUC value for the ENM was very high (0.997), and the predicted range (Fig 4A) represented the species’ current distribution well, except for the east of Alxa Desert, an area of high habitat suitability, was absent (Fig 4B). Range shrinkage in the LGM was showed by CCSM climate models.

## Discussion

### Genetic variation in *Ephedra przewalskii*

Total gene diversity of *Ephedra przewalskii* was high compared with other desert shrubs in northwestern China, such as *Reaumuria soongarica* (*H*_*T*_ = 0.607) and *Ammopiptanthus mongolicus* (*H*_*T*_ = 0.434) [8, 51], and this was consistent with the results of *E*. *przewalskii* in Qin et al. (2013) [11]. *E*. *przewalskii* probably originated in the late Tertiary [52], and the long evolution process in northwestern China has evidently accumulated the degree of genetic diversity [53,54]. *E*. *przewalskii* spread across almost the entire arid zone in northwestern China. The habitats varied in geology and topography, might harbour locally adapted genetic variations of the species. All these might help account for the high level of genetic diversity observed in the species.

High total genetic diversity but low within-population genetic diversity resulted in significant genetic differentiation among populations, which was also supported by AMOVA analyses. Several factors might account for the high level of genetic differentiation among populations and geographic groups. First, numerous geographic barriers within the distribution range probably promote vicariant processes of the species. Populations in Tarim Basin were separated from those in Qaidam Basin by Kunlun Mountains and Altun Mountains, and separated from those in Junggar Basin by the extensive Tianshan Mountains. Thoughout Tarim Basin, populations and groups can be far from each other and separated by a vast desert. The high mountains and large desert expanse might obstruct gene flow and increase heterogeneity among populations and geographic groups. Second, we detected range expansion occurred in some subdivided geographic groups of the species. Repeated founder effect during range expansion could make some alleles fixed in populations, consequently reduced heterozygosity within populations and increased heterogeneity between populations [55,56].

### Allopatric divergence in *Ephedra przewalskii*

As shown by the genetic variation distribution in Fig 1, there were no shared haplotypes between Xinjiang Province and Qaidam Basin. In the phylogeny, haplotypes in Xinjiang clustered into a clade, which was separate from those in Qaidam Basin (Fig 3). In addition, the main genetic variation occurred among populations, geographic regions, and the subdivided geographic groups, as was demonstrated by the AMOVA analysis (Table 2). Together, these data demonstrated a high genetic differentiation among populations, regions, and geographical groups.

In northwestern China, the generally dry climate could date back to the late-Miocene, and aridification promoted the formation of salt mineral resources in Qaidam Basin [57,58]. Since the Pliocene, uplifting of Kunlun Mountains and other ranges of the northern Tibetan Plateau has caused increased aridity in northwestern China because of the growth of rain shadows [59,60,61]. According to Qin et al. (2013) [11], divergence of lineages of *Ephedra* could be dated to the Middle or Late Miocene, and this was very likely linked to the uplift of the QTP and Asian aridification. The climate in northwestern China became cold during the Quaternary [62], and during this time, aridification was usually correlated with periodically cold glacial episodes [50,63]. From the early to middle Pleistocene, the dry climate developed continually, and deserts and gobi further expanded in northwestern China [64]. When it came to the middle Pleistocene, vast Taklimakan Desert has been formed. In Tarim Basin, haplotypes were distinct from the north to the south as well as from the west to the south (Fig 1). The genetic divergence between the north and south could have resulted from Taklimakan Desert in the center of the basin, a vast geographic barrier for gene flow exchanging between the populations on either side of the basin. Groups in the west rim were distant from those in the south of the basin, and the long distances between them could make it difficult for gene exchange. Although haplotype A was shared among Tarim basin, Junggar Basin, and Hexi Corridor, genetic variations in the north and west of Junggar Basin (haplotype H, I, G, J) were absolutely different from those in Tarim Basin and Hexi Corridor. Junggar Basin is separated from Tarim Basin by Tianshan Mountains, and because of the high mountains and deserts barriers, populations in the north and west of Junggar Basin cannot easily exchange genes with those distributed in the west and south of Tarim Basin. Though Junggar Basin is connected to Hexi Corridor by Hami Basin, the long distance between the west and north of Junggar basin and Hexi Corridor would be also a geographic barrier for the gene flow of the populations. For Qaidam Basin, it is separated from Tarim basin and Junggar Basin by Kunlun Mountains, Altun Mountains, and Qilian Mountains, thus the closed topography made gene flow among these basins easily interrupted.

Northwestern China entered into the largest glaciation at approximately 0.8–0.6 Mya, and the climate was much colder and dryer beyond the extent of any former periods [50,64]. Extremely low temperatures during glacial periods was usually correlated with intraspecific differentiation for plant species in allopatric regions, such as *Hippophae tibetana* Schltdl. and *Aconitum gymnandrum* Maxim. distributed in the QTP, with the major lineages diverging in the early Pleistocene as the cooling climate drove speciation [65, 66]. Aridification accompanied with cold temparatures during glacial periods was another driver on intraspecific or specific differentiation, such as *Reaumuria soongarica* (Pall.) Maxim. and *Lagochilus* Bunge ex Benth., distributed in northwestern China [8,67]. The main lineages of *Ephedra przewalskii* (node 1 in Fig 3) diverged approximately 0.73 Mya (early-middle Pleistocene), thus, we infer that the extremely cold and dry climate during this period have resulted in the contraction and fragmentation of the geographic range of the species. Consequently, the intensified barriers for gene flow led to genetic differentiation among Tarim Basin, Junggar Basin, and Qaidam Basin. In addition, the extreme climate in early-middle Pleistocene might also cause extinct events of some genetic variations. Haplotype P (in the south of Hexi Corridor) was closely connected with haplotype D distributed far away in the west of Tarim Basin by two evolution steps in the network. We speculate that intermediate evolution variants between them have been extinct due to hard adaptation to the extreme climate during the long evolution process.

### Range shifts in *Ephedra przewalskii*

Climate oscillation during the Quaternary is usually considered to have profoundly influenced the current geographic distribution patterns of plants [68]. The results of the ENMs suggested that the potential present distribution approximately included the existing sampled locations as well as others areas suitable for living, such as the north of Alxa Desert, from which the populations are currently absent. We speculate this was caused by long-term dispersal barriers over historical time. In CCSM models, the predicted distribution of the species at the LGM was much smaller than present. During the LGM, the climate was cooler and drier than it currently is, and the hostile environment would be expected to force species to suitable habitats, shrinking the distribution to a smaller range.

Although there is no palynological or macro-fossil evidence, there are signatures that support recent range expansion, including the statistically significant Fu’s *F*_*S*_, Tajima’s *D*, and unimodel mismatch distributions. As mentioned, the recent expansion events of the groups occurred during the interglacial period. After the glacial period, temperatures increased and the climate warmed, resulting in a greater amount of glacial ice melting from the surrounding high mountains. The increased run-off infused the basins making the habitats moister [64], which was more suitable for the species to expand their distribution areas into more suitable habitats. During the colonization into a new territory, strong genetic drift would happen in populations on the edge of expansion wave, and some alleles would spread wide areas and reach a high frequency, a process called genetic surfing [69,70]. Consequently the homogenization of the expansion populations would increased, inducing the new occupied areas into distinct sectors from the ancestral population [71,72]. Variants of haplotype A in Tarim Basin, G in Junggar Basin, and M in Qaidam basin might be such cases as this. Haplotype A spread along the north rim of Tarim Basin, and extended north to Junggar Basin and south to Hexi Corridor through Urumqi, Turpan Basin, and Hami Basin. Haplotype M, in contrast, spread into Qaidam Basin, and extended its range east to Hexi Corridor, and further to Alxa Desert. During the multiple processes of colonization, populations with genetic distinction from dispersed refugia would merge with each other, resulting in admixture of the genetic variations from distinct populations [73,74]. Examples of this colonization pattern appear to include: populations in Tarim Basin that have mixed distributions of haplotypes A, E, and F, such as population 21; populations in Junggar Basin that have mixed distributions of haplotypes A and G, such as populations 23–25; and populations in Hexi Corridor and Alxa Desert that have mixed distributions of haplotypes A and M.

## Supporting Information

S1 Fig Mismatch distribution analysis for chloroplast DNA data for (a) group (1) of *Ephedra przewalskii* that includes populations 1–12, 25, 30–31 (SSD = 0, *P* = 0.39), (b) group (6) of *E*. *przewalskii* that includes populations 22–24, 27–29 (SSD = 0.0002, *P* = 0.39), (c) group (10) of *E*. *przewalskii* that includes populations 36–44 (SSD = 0.0001, *P* = 0.33), and (d) all samples of *N*. *roborowskii* (SSD = 0.0105, *P* = 0.13).
(EPS)

S1 Table Details of sample locations, sample size, and haplotype frequencies for 45 populations of *Ephedra przewalskii*. Figures in the parenthesis represent the number of the haplotypes.
(DOC)

S2 Table Sixteen haplotypes of *Ephedra przewalskii* recognized on basis of two chloroplast DNA sequences, *trn*H*—psb*A and *trn*T–*trn*F. (DOC)

## References

[1] Dang RL, Pan XL, Gu XF (2002) Floristic analysis of spermatophyte genera in the arid deserts area in North-West China. Guihaia 22(2): 121–128.

[2] Sun JM, Ye J, Wu WY, Ni XJ, Bi SD, Zhang ZQ, et al (2010) Late Oligocene–Miocene mid-latitude aridification and wind patterns in the Asian interior. Geology 38: 515–518.

[3] Li WC. The Chinese Quaternary vegetation and environment Beijing: Science Press; 1998.

[4] Ma SM, Zhang ML, Sanderson SC (2012) Phylogeography of the rare *Gymnocarpos przewalskii* (Caryophyllaceae):indications of multiple glacial refugia in north-western China. Australian Journal of Botany 60: 20–31.

[5] Su ZH, Zhang ML (2013) Evolutionary response to Quaternary climate aridification and oscillations in northwestern China revealed by chloroplast phylogeography of *Nitraria sphaerocarpa* (Nitrariaceae). Biological Journal of the Linnean Society 109: 757–770.

[6] Guo YP, Zhang R, Chen CY, Zhou DW, Liu JQ (2010) Allopatric divergence and regional range expansion of *Juniperus sabina* in China. Journal of Systematics and Evolution 48: 153–160.

[7] Zhang HX, Zhang ML (2012) Identifying a contact zone between two phylogeographic lineages of *Clematis sibirica* (Ranunculeae) in the Tianshan and Altai Mountains. Journal of Systematics and Evolution 50: 295–304.

[8] Li ZH, Chen J, Zhao GF, Guo YP, Kou YX, Ma YZ, et al (2012) Response of a desert shrub to past geological and climatic change: a phylogeographic study of *Reaumuria soongarica* (Tamaricaceae) in western China. Journal of Systematics and Evolution 50: 351–361.

[9] Su ZH, Zhang ML, Cohen JI (2012) Phylogeographic and demographic effects of Quaternary climate oscillations in *Hexinia polydichotoma* (Asteraceae) in Tarim Basin and adjacent areas. Plant Systematics and Evolution 298: 1767–1776.

[10] Fu LG, Yu YF, Harald R. Ephedraceae In: Wu Z. Y., & Raven P. H. (Eds.) Flora of China, vol 4. Beijing: Science Press; 1999 pp. 98.

[11] Qin AL, Wang MM, Cun YZ, Yang FS, Wang SS, Ran JH, et al (2013) Phylogeographic evidence for a link of species divergence of *Ephedra* in the Qinghai-Tibetan Plateau and adjacent regions to the Miocene Asian Aridification. PLoS One 8(2): e56243 10.1371/journal.pone.0056243 23418542 PMC3571962

[12] Clegg MT, Zurawski G. Chloroplast DNA and the Study of Plant Phylogeny: Present Status and Future Prospects In: Soltis P. S., Soltis D. E., Doyle J. J. (Eds.) Molecular Systematics of Plants. Chapman & Hall, New York, NY, 1992 pp. 2.

[13] Comes HP, Kadereit JW (1998) The effect of quaternary climatic changes on plant distribution and evolution. Trends in Plant Science 3: 432–438.

[14] Avise JC. Phylogeography: the history and formation of species. Harvard University Press; 2000.

[15] Soltis DE, Morris A, McLachlan JS, Manos PS, Soltis PS (2006) Comparative phylogeography of unglaciated eastern North America. Molecular Ecology 15: 4261–4293. 17107465 10.1111/j.1365-294X.2006.03061.x

[16] Liu JQ, Sun YS, Ge XJ, Gao LM, Qiu YX (2012) Phylogeographic studies of plants in China: advances in the past and directions in the future. Journal of Systematics and Evolution 50: 267–275.

[17] Petit RJ, Aguinagalde I, de Beaulieu JL, Bittkau C, Brewer S, Cheddadi R, et al (2003) Glacial refugia: hotspots but not melting pots of genetic diversity. Science 300: 1563–1565. 12791991 10.1126/science.1083264

[18] Phillips SJ, Anderson RP, Schapire RE (2006) Maximum entropy modeling of species geographic distributions. Ecological Modelling 190: 231–259.

[19] Alsos IG, Alm T, Normand S, Brochmann C (2009) Past and future range shifts and loss of diversity in dwarf willow (*Salix herbacea* L.) inferred from genetics, fossils and modelling. Global Ecology and Biogeography 18: 223–239.

[20] Marske KA, Leschen RAB, Buckley TR (2011) Reconciling phylogeography and ecological niche models for New Zealand beetles: looking beyond glacial refugia. Molecular Phylogenetics and Evolution 59: 89–102. 10.1016/j.ympev.2011.01.005 21262367

[21] Rogers SO, Bendich AJ (1985) Extraction of DNA from milligram amounts of fresh, herbarium and mummified plant-tissues. Plant Molecular Biology 5: 69–76. 10.1007/BF00020088 24306565

[22] Doyle JJ, Doyle JL (1987) A rapid DNA isolation procedure from small quantities of fresh leaf tissues. Phytochemical Bulletin 19: 11–15.

[23] Sang T, Crawford DJ, Stuessy TF (1997) Chloroplast DNA phylogeny, reticulate evolution, and biogeography of *Paeonia* (Paeoniaceae). American Journal of Botany 84: 1120–1136. 21708667

[24] Taberlet P, Gielly L, Pautou G, Bouvet J (1991) Universal primers for amplification of three non-coding regions of chloroplast DNA. Plant Molecular Biology 17: 1105–1109. 1932684 10.1007/BF00037152

[25] Thompson JD, Higgins DG, Gibson TJ (1994) Clustal-W–improving the sensitivity of progressive multiple sequence alignment through sequence weighting, position-specific gap penalties and weight matrix choice. Nucleic Acids Research 22: 4673–4680. 7984417 10.1093/nar/22.22.4673 PMC308517

[26] Swofford DL (2002) PAUP*: phylogenetic analysis using parsimony (and other methods), Version 4.0b10. Sunderland: Sinauer Associates.

[27] Posada D, Crandall KA (1998) Modeltest: testing the model of DNA substitution. Bioinformatics 14: 817–818. 9918953 10.1093/bioinformatics/14.9.817

[28] Drummond AJ, Nicholls GK, Rodrigo AG, Solomon W (2002) Estimating mutation parameters, population history and genealogy simultaneously from temporally spaced sequence data. Genetics 161: 1307–1320. 12136032 10.1093/genetics/161.3.1307 PMC1462188

[29] Drummond AJ, Rambaut A (2007) BEAST: Bayesian evolutionary analysis by sampling trees. BMC Evolutionary Biology 7: 214 17996036 10.1186/1471-2148-7-214 PMC2247476

[30] Simmons MP, Ochoterena H (2000) Gaps as characters in sequence based phylogenetic analyses. Systematic Biology 49: 369–381. 12118412

[31] Wolfe KH, Li WH, Sharp PM (1987) Rates of nucleotide substitution vary greatly among plant mitochondrial, chloroplast, and nuclear DNAS. Proceedings of The National Academy of Sciences of The United States 84: 9054–9058. 10.1073/pnas.84.24.9054 PMC299690 3480529

[32] Jia DR, Abbott RJ, Liu TL, Mao KS, Bartish IV, Liu JQ (2012) Out of the Qinghai–Tibet Plateau: evidence for the origin and dispersal of Eurasian temperate plants from a phylogeographic study of *Hippophaë rhamnoides* (Elaeagnaceae). New Phytologist 194: 1123–1133. 10.1111/j.1469-8137.2012.04115.x 22432741

[33] Templeton AR, Crandall KA, Sing CF (1992) A cladistic analysis of phenotypic associations with haplotypes inferred from restriction endonuclease mapping and DNA sequence data. III. Cladogram estimation. Genetics 132: 619–633. 1385266 10.1093/genetics/132.2.619 PMC1205162

[34] Clement M, Posada D, Crandall KA (2000) TCS: a computer program to estimate gene genealogies. Molecular Ecology 9: 1657–1659. 11050560 10.1046/j.1365-294x.2000.01020.x

[35] Dupanloup I, Schneider S, Excoffier L (2002) A simulated annealing approach to define the genetic structure of populations. Molecular Ecology 11: 2571–2581. 12453240 10.1046/j.1365-294x.2002.01650.x

[36] Excoffier L, Smouse P, Quattro J (1992) Analysis of molecular variance inferred from metric distances among DNA haplotypes: applications to human mitochondrial DNA restriction data. Genetics 131: 479–491. 1644282 10.1093/genetics/131.2.479 PMC1205020

[37] Tajima F (1989) Statistical method for testing the neutral mutation hypothesis by DNA polymorphism. Genetics 123: 585–595. 2513255 10.1093/genetics/123.3.585 PMC1203831

[38] Fu YX (1997) Statistical tests of neutrality of mutations against population growth, hitchhiking, and background selection. Genetics 147: 915–925. 9335623 10.1093/genetics/147.2.915 PMC1208208

[39] Jaeger JR, Riddle BR, Bradford DF (2005) Cryptic neogene vicariance and Quaternary dispersal of the redspotted toad (*Bufo punctatus*): insights on the evolution of North American warm desert biotas. Molecular Ecology 14: 3033–3048. 16101772 10.1111/j.1365-294X.2005.02645.x

[40] Smith CI, Farrell BD (2005) Range expansions in the flightless longhorn cactus beetles, *Moneilema gigas* and *Moneilema armatum*, in response to Pleistocene climate changes. Molecular Ecology 14: 1025–1044. 15773934 10.1111/j.1365-294X.2005.02472.x

[41] Aris-Brosou S, Excoffier L (1996) The impact of population expansion and mutation rate heterogeneity on DNA sequence polymorphism. Molecular Biology and Evolution 13: 494–504. 8742638 10.1093/oxfordjournals.molbev.a025610

[42] Tajima F (1996) The amount of DNA polymorphism maintained in a finite population when the neutral mutation rate varies among sites. Genetics 143: 1457–1465. 8807315 10.1093/genetics/143.3.1457 PMC1207412

[43] Slatkin M, Hudson RR (1991) Pairwise comparisons of mitochondrial DNA sequences in stable and exponentially growing populations. Genetics 129: 555–562. 1743491 10.1093/genetics/129.2.555 PMC1204643

[44] Rogers A, Harpending H (1992) Population growth makes waves in the distribution of pairwise genetic differences. Molecular Biology and Evolution 9: 552–569. 1316531 10.1093/oxfordjournals.molbev.a040727

[45] Hijmans RJ, Cameron SE, Parra JL, Jones PG, Jarvis A (2005) Very high resolution interpolated climate surfaces for global land areas. International Journal of Climatology 25: 1965–1978.

[46] Collins WD, Bitz CM, Blackmon ML, Bonan GB, Bretherton CS, Carton JA, et al (2006) The Community Climate System Model version 3 (CCSM3). Journal of Climate 19: 2122–2143.

[47] Peterson AT, Nakazawa Y (2008) Environmental data sets matter in ecological niche modelling: an example with *Solenopsis invicta* and *Solenopsis richteri*. Global Ecology and Biogeography 17: 135–144.

[48] Phillips SJ, Dudík M (2008) Modeling of species distributions with Maxent: new extensions and a comprehensive evaluation. Ecography 31: 161–175.

[49] Swets JA (1988) Measuring the accuracy of diagnostic systems. Science 240: 1285–1293. 3287615 10.1126/science.3287615

[50] Shi YF, Cui ZJ, Su Z. The Quaternary glaciations and environmental variations in China Hebei: Hebei Science and Technology Publishing House; 2005 pp. 99–100.

[51] Su ZH, Pan BR, Zhang ML, Shi W (2016) Conservation genetics and geographic patterns of genetic variation of endangered shrub *Ammopiptanthus* (Fabaceae) in northwestern China. Conservation Genetics 17: 485–496.

[52] Wu ZY, Sun H, Zhou ZK, Li DZ, Peng H. Floristics of seed plants from China Beijing: Science Press; 2010.

[53] Chiang TY, Schaal BA (1999) Phylogeography of North American populations of the moss species *Hylocomium splendens* based on the nucleotide sequence of internal transcribed spacer 2 of nuclear ribosomal DNA. Molecular Ecology 8: 1037–1042.

[54] Huang S, Chiang YC, Schaal BA, Chou CH, Chiang TY (2001) Organelle DNA phylogeography of *Cycas taitungensis*, a relict species in Taiwan. Molecular Ecology 10: 2669–2681. 11883881 10.1046/j.0962-1083.2001.01395.x

[55] Austerlitz F, Jung-Muller B, Godelle B, Gouyon PH (1997) Evolution of coalescence times, genetic diversity and structure during colonization. Theoretical Population Biology 51: 148–164.

[56] Edmonds CA, Lillie AS, Cavalli-Sforza LL (2004) Mutations arising in the wave front of an expanding population. Proceedings of the National Academy of Sciences of the United States of America 101: 975–979. 14732681 10.1073/pnas.0308064100 PMC327127

[57] Fang XM, Xi XX, Li JJ, Mu DF (1997) The discover and meaning of the drying climate in the Late Miocene in northwest China. Chinese Science Bulletin 42(23): 2521–2524.

[58] Xi XX, Mu DF, Fang XM, Li JJ (1998) Climatic change since the Late Miocene in west China: evidence from anion Chlorine in the Linxia Red Basin. Acta Sedimentologica Sinica 16(2): 155–160.

[59] Zheng HB, Powell CM, Butcher K, Cao JJ (2003) Late Neogene loess deposition in southern Tarim Basin: tectonic and palaeoenvironmental implications. Tectonophysics 375: 49–59.

[60] Sun JM, Zhang LY, Deng CL, Zhu RX (2008) Evidence for enhanced aridity in the Tarim Basin of China since 5.3 Ma. Quaternary Science Reviews 27: 1012–1023.

[61] Miao YF, Herrmann M, Wu FL, Yan XL, Yang SL (2012) What controlled Mid-Late Miocene long-term aridification in Central Asia?–global cooling or Tibetan Plateau uplift: a review. Earth-Science Reviews 112: 155–172.

[62] Guo ZT, Peng SZ, Hao QZ, Chen XH, Liu TS (1999) Late Tertiary development of aridification in northwestern China: link with the arctic ice-sheet formation and Tibetan uplifts. Quaternary Science 6: 556–566. (in Chinese with English abstract).

[63] Xu X, Kleidon A, Miller L, Wang SQ, Wang LQ, Dong GC (2010) Late Quaternary glaciation in the Tianshan and implications for palaeoclimatic change: a review. Boreas 39: 215–232.

[64] Williams MAJ, Dunkerley DL, De Dekker P, Kershaw AP, Stokes T. Quaternary environments. London: Edward Arnold; 1993.

[65] Wang LY, Abbott RJ, Zhang W, Chen P, Wang YJ, Liu JQ (2009) History and evolution of alpine plants endemic to the Qinghai–Tibetan Plateau: *Aconitum gymnandrum* (Ranunculaceae). Molecular Ecology 18: 709–721. 10.1111/j.1365-294X.2008.04055.x 19175501

[66] Jia DR, Liu TL, Wang LY, Zhou DW, Liu JQ (2011) Evolutionary history of an alpine shrub *Hippophae tibetana* (Elaeagnaceae): allopatric divergence and regional expansion. Biological Journal of the Linnean Society 102: 37–50.

[67] Meng HF, Gao XY, Huang JF, Zhang ML (2015) Plant phylogeography in arid Northwest China: Retrospectives and perspectives. Journal of Systematics and Evolution 53: 33–46.

[68] Hewitt GM (2004) Genetic consequences of climatic oscillations in the Quaternary. Proceedings of the Royal Society of London. Series B-Biological Sciences 359: 183–195. 10.1098/rstb.2003.1388 PMC1693318 15101575

[69] Klopfstein S, Currat M, Excoffier L (2006) The fate of mutations surfing on the wave of a range expansion. Molecular Biology and Evolution 23: 482–490. 16280540 10.1093/molbev/msj057

[70] Excoffier L, Foll M, Petit RJ (2009) Genetic consequences of range expansions. Annual Review of Ecology, Evolution, and Systematics 40: 481–501.

[71] Excoffier L, Ray N (2008) Surfing during population expansions promotes genetic revolutions and structuration. Trends in Ecology & Evolution 23: 347–351. 18502536 10.1016/j.tree.2008.04.004

[72] Excoffier L, Foll M, Petit RJ (2009) Genetic Consequences of Range Expansions. Annual Review of Ecology, Evolution, and Systematics 40: 481–501.

[73] Nichols RA, Hewitt GM (1994) The genetic consequences of long distance dispersal during colonization. Heredity 72: 312–317.

[74] Mellick R, Lowe A, Allen C, Hill RS, Rossetto M (2012) Palaeodistribution modelling and genetic evidence highlight differential post-glacial range shifts of a rain forest conifer distributed across a latitudinal gradient. Journal of Biogeography 39: 2292–2302.

