# Supplementary material for: Evolutionary History of a Desert Shrub Ephedra przewalskii (Ephedraceae): Allopatric Divergence and Range Shifts in Northwestern China
Source: PLoS One. 2016 Jun 28;11(6):e0158284. doi: 10.1371/journal.pone.0158284 (PMC4924853; doi:10.1371/journal.pone.0158284)
Supplement: S1 Table — Figures in the parenthesis represent the number of the haplotypes. (DOC) [file pone.0158284.s002.doc]

**S1 Table.** Details of sample locations, sample size, and haplotype frequencies for 45 populations of *Ephedra przewalskii*. Figures in the parenthesis represent the number of the haplotypes.

| Region | Number | Location | Latitude | Longitude | Altitude | cp |
| --- | --- | --- | --- | --- | --- | --- |
|  |  |  | (N) | (E) | (m) | Haplotype |
| Urumqi | 1 | Yanhu | 43.30 | 88.35 | 1009 | A(10) |
| Turpan Basin | 2 | Tuokexun | 42.48 | 88.53 | 1598 | A(10) |
| Tarim Basin | 3 | Kumush | 42.24 | 88.18 | 937 | A(11) |
|  | 4 | Heshuo | 42.22 | 87.82 | 1498 | A(11) |
|  | 5 | Wushitala | 42.23 | 87.43 | 1262 | A(11) |
|  | 6 | Tashidian | 41.87 | 86.34 | 1203 | A(10);B(1);C(1) |
|  | 7 | Luntai | 41.95 | 84.15 | 1090 | A(11) |
|  | 8 | Kuqa | 41.76 | 83.39 | 1190 | A(10) |
|  | 9 | Yanshuigou | 41.85 | 82.77 | 1320 | A(10) |
|  | 10 | Tiereke | 41.85 | 81.65 | 1457 | A(10) |
|  | 11 | Chaerqi | 41.57 | 81.25 | 1454 | A(10) |
|  | 12 | Keping | 40.69 | 79.82 | 1455 | A(12) |
|  | 13 | Patrul | 39.96 | 78.44 | 1135 | D(10) |
|  | 14 | Atushi | 39.73 | 76.20 | 1295 | D(10) |
|  | 15 | Mayikake | 39.59 | 75.10 | 1818 | D(10) |
|  | 16 | Wuheshalu | 39.66 | 74.75 | 2290 | D(8);A(2) |
|  | 17 | Pishan | 37.58 | 78.18 | 1424 | E(10) |
|  | 18 | Cele | 36.97 | 80.82 | 1407 | E(10) |
|  | 19 | Qiemo1 | 38.71 | 87.10 | 1355 | E(3);F(7) |
|  | 20 | Qiemo2 | 38.03 | 85.59 | 1362 | E(4);F(6) |
|  | 21 | Ruoqiang | 39.00 | 88.15 | 889 | A(2);E(4);F(4) |
| Junggar Basin | 22 | Qitai1 | 44.25 | 89.63 | 710 | G(11) |
|  | 23 | Beitashan | 44.60 | 90.35 | 600 | A(2);G(8) |
|  | 24 | Qitai2 | 44.27 | 90.13 | 712 | A(1);G(9) |
|  | 25 | Karamay | 45.63 | 84.84 | 294 | A(2);G(4);H(1);I(3) |
|  | 26 | Wuchang | 45.89 | 85.36 | 291 | A(2);I(9) |
|  | 27 | Alashankou | 45.10 | 82.56 | 286 | G(11) |
|  | 28 | Hefeng | 46.24 | 85.86 | 548 | G(10) |
|  | 29 | Burqin | 47.65 | 86.92 | 494 | G(9);J(1) |
| Hami Basin | 30 | Hami | 42.91 | 93.62 | 862 | A(10) |
| Hexi  Corridor | 31 | Liuyuan | 41.17 | 95.38 | 1893 | A(9);K(2) |
|  | 32 | Guazhou | 40.39 | 95.67 | 1156 | A(6);L(1);M(4) |
|  | 33 | Jinta | 39.97 | 98.92 | 1242 | A(2);M(8) |
| Alxa  Desert | 34 | Ejina1 | 40.35 | 99.81 | 1009 | A(3);M(7) |
|  | 35 | Ejina2 | 41.87 | 100.63 | 963 | A(2);M(8) |
| Hexi  Corridor | 36 | Yumen | 40.22 | 97.06 | 1573 | M(10) |
|  | 37 | Akesai | 39.67 | 94.36 | 2703 | M(10) |
|  | 38 | Subei | 39.74 | 94.57 | 1101 | M(10);N(1) |
| Qaidam  Basin | 39 | Lvcaoshan | 37.58 | 95.68 | 3283 | M(10) |
|  | 40 | Mahuanggou1 | 37.43 | 95.40 | 3184 | M(9);O(2) |
|  | 41 | Mahuanggou2 | 37.20 | 95.50 | 3095 | M(8);O(2) |
|  | 42 | Daqaidam | 37.95 | 95.10 | 3267 | M(12) |
|  | 43 | Golmud | 36.29 | 94.80 | 2883 | M(10) |
|  | 44 | Ruomuhong | 36.37 | 96.42 | 2972 | M(12) |
| Hexi  Corridor | 45 | Xuebai | 38.57 | 102.86 | 1378 | P(10) |
