# Supplementary material for: Evolutionary History of a Desert Shrub Ephedra przewalskii (Ephedraceae): Allopatric Divergence and Range Shifts in Northwestern China
Source: PLoS One. 2016 Jun 28;11(6):e0158284. doi: 10.1371/journal.pone.0158284 (PMC4924853; doi:10.1371/journal.pone.0158284)
Supplement: S2 Table — (DOC) [file pone.0158284.s003.doc]

**S2 Table.** Sixteen haplotypes of *Ephedra przewalskii* recognized on basis of two chloroplast DNA sequences, *trn*H *- psb*A and *trn*T–*trn*F.

|  | Sequence position | | | | | | | | | | | |
| --- | --- | --- | --- | --- | --- | --- | --- | --- | --- | --- | --- | --- |
|  | 4 | 4 | 4 | 4 | 4 | 5 | 7 | 9 | 1 | 1 | 1 | 1 |
|  | 2 | 4 | 6 | 7 | 9 | 9 | 3 | 9 | 0 | 0 | 0 | 1 |
|  | 4 | 3 | 7 | 0 | 3 | 6 | 0 | 6 | 3 | 9 | 9 | 1 |
|  |  |  |  |  |  |  |  |  | 2 | 3 | 4 | 7 |
| Haplotype |  |  |  |  |  |  |  |  |  |  |  |  |
| A | T | G | C | A | T | G | C | A | A | G | T | G |
| B | - | G | C | A | T | G | C | A | A | G | T | - |
| C | - | G | C | A | T | G | C | A | A | G | T | G |
| D | T | G | C | A | A | G | C | A | A | G | T | G |
| E | T | G | C | A | A | G | T | A | A | G | T | G |
| F | - | G | C | A | A | G | T | A | A | G | T | G |
| G | T | G | C | A | T | G | C | A | C | G | T | G |
| H | T | G | G | A | T | G | C | A | A | G | T | G |
| I | T | G | C | A | T | G | C | T | A | G | T | G |
| J | - | G | C | A | T | G | C | A | C | G | T | G |
| K | T | G | C | - | T | G | C | A | A | G | T | G |
| L | T | G | C | A | T | A | C | A | A | G | T | G |
| M | T | G | C | A | A | A | C | A | A | G | T | G |
| N | T | T | C | A | A | A | C | A | A | G | T | G |
| O | - | G | C | A | A | A | C | A | A | G | T | G |
| P | T | G | C | A | A | G | C | A | A | - | - | G |
